# Integrated Management of Childhood Illness implementation in Nepal: understanding strategies, context, and outcomes

**DOI:** 10.1186/s12887-023-03889-3

**Published:** 2024-02-28

**Authors:** Raj Kumar Subedi, Amelia VanderZanden, Kriti Adhikari, Sasmrita Bastola, Lisa R. Hirschhorn, Agnes Binagwaho, Mahesh Maskey

**Affiliations:** 1 Nepal Public Health Foundation, Kathmandu, Nepal; 2 University of Global Health Equity, Kigali, Rwanda; 3 Northwestern University Feinberg School of Medicine, Chicago, USA

**Keywords:** Implementation research, Integrated management of childhood illness, Under-5 mortality, Implementation strategies, Nepal

## Abstract

**Background:**

Health system-delivered evidence-based interventions (EBIs) are important to reducing amenable under-5 mortality (U5M). Implementation research (IR) can reduce knowledge gaps and decrease lags between new knowledge and its implementation in real world settings. IR can also help understand contextual factors and strategies useful to adapting EBIs and their implementation to local settings. Nepal has been a leader in dropping U5M including through adopting EBIs such as integrated management of childhood illness (IMCI). We use IR to identify strategies used in Nepal’s adaptation and implementation of IMCI.

**Methods:**

We conducted a mixed methods case study using an implementation research framework developed to understand how Nepal outperformed its peers between 2000–2015 in implementing health system-delivered EBIs known to reduce amenable U5M. We combined review of existing literature and data supplemented by 21 key informant interviews with policymakers and implementers, to understand implementation strategies and contextual factors that affected implementation outcomes. We extracted relevant results from the case study and used explanatory mixed methods to understand how and why Nepal had successes and challenges in adapting and implementing one EBI, IMCI.

**Results:**

Strategies chosen and adapted to meet Nepal’s specific context included leveraging local research to inform national decision-makers, pilot testing, partner engagement, and building on and integrating with the existing community health system. These cross-cutting strategies benefited from facilitating factors included community health system and structure, culture of data use, and local research capacity. Geography was a critical barrier and while substantial drops in U5M were seen in both the highest and lowest wealth quintiles, with the wealth equity gap decreasing from 73 to 39 per 1,000 live births from 2001 to 2016, substantial geographic inequities remained.

**Conclusions:**

Nepal’s story shows that implementation strategies that are available across contexts were key to adopting and adapting IMCI and achieving outcomes including acceptability, effectiveness, and reach. The value of choosing strategies that leverage facilitating factors such as investments in community-based and facility-based approaches as well as addressing barriers such as geography are useful lessons for countries working to accelerate adaptation and implementation of strategies to implement EBIs to continue achieving child health targets.

## Background

Over the past several decades, a surge of proven evidence-based interventions (EBIs) known to reduce under-5 mortality (U5M) has led to important progress in reducing U5M especially in low- and middle-income countries (LMICs) [1–3]. Yet countries’ goals of reducing those child deaths preventable by a quality and effective system have been slowed by the lag between scientific progress and the dissemination and implementation at scale of these EBIs with quality and equity [4–6]. There is continued need to increase understanding of how existing and emerging EBIs in geographically diverse and complex settings were implemented, successfully or otherwise, in order to learn from and generalize this experience in other contexts [7, 8].

Implementation research (IR) studies how EBIs are adopted and integrated into community and clinical settings to improve individual and population health [9]. This work bridges the gap between what we know about the effectiveness of EBIs and their actual uptake into a health system [10]. IR facilitates understanding of the successes and challenges in implementing EBIs in LMICs and is crucial to understanding how countries are successful in taking up new innovations as new EBIs emerge [8, 11]. Yet the literature on EBIs tends to focus on effectiveness and coverage. Often, it does not capture important insights into what was actually done – implementation strategies – and what worked and why, including the barriers and facilitators – contextual factors – which may have influenced strategy choice and success [12]. As there is increasing evidence on adapting existing EBIs and integrating new EBIs, it is critical to understand how countries succeed in accelerating the uptake and integration of new innovations into their health systems, thereby narrowing the gap between research and practice [13].

To bridge this gap, between 2017 and 2020 we conducted implementation research case studies in six countries (Nepal, Rwanda, Senegal, Bangladesh, Peru, and Ethiopia) which from 2000–2015 outperformed their geographic and economic peers in reducing amenable U5M – deaths preventable through health system-delivered EBIs (the full Nepal case study is available at https://www.exemplars.health/topics/under-five-mortality/nepal). We developed a hybrid implementation research framework building on existing frameworks, to guide the study in identifying implementation strategies, contextual factors which served as barriers or facilitators in LMICs relevant to amenable U5M, and implementation outcomes such as acceptability, effectiveness, and fidelity (Appendix 1) [14].

Nepal has experienced challenges to reducing U5M related to geography, conflict, and deeply ingrained social inequities. The country witnessed a decade-long political conflict (1996–2006) which resulted in significant loss of lives, internal displacement, and economic loss [15]. The country’s mountainous terrain makes it difficult to deliver health services in the community [16]. Further, longstanding social inequities including unequal access to economic, social, and political representation across the country’s many ethnic groups have continued to persist despite efforts to put an end to them [17]. Yet between 2001 and 2016, Nepal saw a marked decline in U5M from 91 deaths per 1,000 live births to 39 deaths per 1,000 live births, a reduction of 57% [18]. Neonatal mortality dropped by 46% (from 39 deaths per 1,000 live births to 21 per 1,000 live births). While the decline in neonatal mortality was slower than that of U5M, both dropped faster than the average in the Southeast Asian Region (47% and 37%, respectively) for the period 2000 to 2015 [19] as well as the average decline across LMICs (44% and 36%) [20]. Important to this success was the exploration, adaptation, and integration of EBIs.

In this paper, we use IR to understand the steps that Nepal took to explore, prepare, implement, adapt, and sustain the implementation of integrated management of childhood illness (IMCI) as an example of one EBI important for U5M reduction, and the strategies adapted and adopted at each step of the process. We identify contextual factors that facilitated or hindered the adoption of IMCI and broader EBI implementation work to better understand how implementation strategies were selected or adapted and the context in which Nepal’s leaders made decisions. Understanding how Nepal was able to overcome barriers and adopt and adapt implementation strategies to implement EBIs such as IMCI can help to provide key lessons that can be replicated in other settings working to accelerate their efforts to implement or adapt new and existing EBIs as new evidence emerges through research and experience.

## Methods

### Study design

We used mixed methods implementation research informed by a hybrid implementation research framework we developed to understand implementation of EBIs in LMICs, drawing from existing frameworks described in detail elsewhere (Appendix 1) [14]. The framework centers a five-step implementation process modified from Aarons et al. which includes Exploration, Preparation, Implementation, Adaptation, and Sustainment (EPIAS). We included contextual factors associated with national, subnational, and community implementation adapted from the Consolidated Framework for Implementation Research and other publications on factors related to U5M, to reflect factors associated with national implementation in LMICs [14, 21–23]. We used the framework to guide our prioritization and interpretation of the research, and to form themes from our findings.

### Evidence-based interventions

We reviewed literature and guidelines from the Millennium Development Goal (MDG) efforts to identify EBIs that existed or were introduced during the study period known to reduce the most common causes of amenable U5M among infants and children in LMICs. This list of EBIs guided both the evidence review and the selection of key informants, as well as our exploration of implementation strategies, contextual factors, and implementation outcomes [14, 24, 25] (Appendix 2).

We focused on two closely related EBIs, facility-based IMCI and community-based IMCI, which Nepal began to implement as a single, integrated intervention (IMCI) beginning in 1999 [26]. We chose IMCI to illustrate the path that Nepal took to identify barriers and facilitators and strategies to address or leverage these contextual factors.

### Data collection

#### Desk review

We conducted a search of available peer-reviewed literature focusing on uptake and implementation of EBIs including IMCI. We used MEDLINE (PubMed) and Google Scholar with search terms including “child mortality” or “under-5 mortality” and “Nepal,” specific EBIs (e.g. “community-based integrated management of childhood illness”), causes of death (e.g. “diarrhea”), and contextual factors identified as relevant to the EBI implementation (e.g. “community health worker programs”). We also looked at sources including national and other reports and policies. We reviewed the documents for implementation strategies, contextual factors, policies, and coverage of the amenable U5M-targeted health system-delivered EBIs in Nepal between 2000 and 2015. We progressively supplemented these results through bibliography review and as additional sources (reports, published articles, case studies) were identified throughout the study process.

#### Quantitative data

We used existing data sources including from the Nepal Demographic and Health Survey (DHS, 2001–2016) and the Institute for Health Metrics and Evaluation (IHME, University of Washington) to extract quantitative data on changing causes of death, mortality rates, EBI coverage, reports on childhood illness, and measures of equity [18, 27, 28].

#### Key informant interviews

We purposively sampled 21 key informants (KIs) involved in EBI implementation between 2000 and 2015 including policymakers and implementers at the national (Ministry of Health), district, and community levels, and key individuals from non-governmental, multilateral, and donor organizations involved in partner-led or -supported activities. The selection of KIs was not intended for saturation but to cover EBIs such as IMCI and was limited by time and resources. The interviews used a semi-structured guide based on the implementation research framework and were designed to understand EBI implementation process, from exploration and preparation, through implementation, adaptation, and sustainment (EPIAS) [14]. Interviews were led by one of the project principal investigators (LRH), the research and project coordinator, or a member of the collaborating team from Nepal Public Health Foundation, with one to two notetakers.

### Analysis

We used a sequential explanatory mixed methods approach, using coverage data to inform qualitative questions [29] and using directed content analysis of the KI interviews based on the EPIAS framework [30, 31]. We identified implementation strategies, contextual factors, and implementation outcomes as available from interviews and the desk review. Using the framework, we created an initial set of codes for EBIs, contextual factors, and implementation outcomes. We coded manually and added new codes as we identified new concepts, contextual factors, or implementation strategies. Guided by the framework we also extracted evidence from the quantitative and qualitative data sources for implementation outcomes including appropriateness, acceptability, feasibility, effectiveness, coverage or reach, and equity where available, to understand the successes or challenges of EBI implementation.

We analyzed and synthesized the findings and presented them for review during a convening of stakeholders in Nepal for feedback and validation, as well as to identify transferable lessons and strategies which have the potential to be adapted and adopted in other countries looking to learn from Nepal’s successes and challenges to accelerate their own work to decrease U5M.

### Ethics

The work was approved by and conducted with the support of the Ministry of Health and the Nepal Health Research Council (approval number 165–2018). Informed consent was obtained from all interview participants. The overall project was reviewed by the Rwanda National Ethics Committee and Northwestern University and determined to be non-human-subjects research.

## Results

Integrated management of childhood illness was chosen as it was designed to treat children for diseases responsible for the leading amenable causes of death: in 2000, acute respiratory infections (ARI), diarrhea, and malnutrition combined were accountable for more than one-third of under-5 deaths in Nepal [32]. The country began piloting a combined facility- and community-based IMCI in districts between 1999–2000 and it was rolled out nationally to all 75 districts by 2009. Care-seeking for children under 5 – a marker of the EBI’s acceptability – between 2001 and 2016 increased: for example, for diarrhea, rising from 21 to 64%; for fever, rising from 24 to 80% [18, 27] (Appendix 3). There was variability in equity of improvements based on geography. While care-seeking for diarrhea increased in both urban and rural settings from 2001 to 2016, there was a larger increase in rural areas (from 21% to 70%, up 49 points) than urban (from 23% to 60%, up 37 points) [18, 27]. There were also increases in care-seeking by wealth category. For example, care-seeking for fever rose from 19% and 33% among the lowest and highest quintiles in 2001 to 59% and 84% in 2016 – but the absolute gap between the quintiles also grew during this period [18, 27].

### Understanding steps and strategies for the implementation of facility- and community-based integrated management of childhood illness

We describe the process for how Nepal chose, adapted, and implemented IMCI including implementation strategies and facilitating and hindering contextual factors which emerged during the five steps outlined in the implementation research framework (Exploration, Preparation, Implementation, Adaptation, and Sustainment) (Table 1).

**Table 1:** Implementation strategies for community-based and facility-based integrated management of childhood illness in Nepal

**Implementation strategy**	**Facility-based IMCI**	**Community-based IMCI**
***Adaptation***	***X***	***X***
Adaptation of existing training and guidelines to reflect local context	**x**	**x**
Expansion and adaptation of existing programs	**x**	**x**
***Data use***	***X***	***X***
Data driven adaptations for cost and feasibility	**x**	**x**
Piloting	**x**	**x**
Local research		**x**
Strengthening management and monitoring and evaluation	**x**	
***Engagement***	***X***	***X***
Engagement of community and local stakeholders	**x**	**x**
Engagement of partners in preparation and for implementation	**x**	**x**
Engagement across sectors	**x**	
Engagement of traditional healers		**x**
***Integration***	***X***	***X***
Integration of program into existing structures	**x**	**x**
Integration into existing community worker capacity		**x**
Integrating monitoring and evaluation with supervision		**x**
Integration of supervision into existing district health offices	**x**	
***Training***	***X***	***X***
Facility staff trained in management and monitoring and evaluation		**x**
Initial and ongoing training through refresher courses		**x**
Training of trainers and cascade to district control and responsibility	**x**	

*IMCI* Integrated management of childhood illness

Throughout this pathway, we identified a number of strategies used by the Government of Nepal to implement or strengthen IMCI. These strategies were chosen and adapted to meet the specific context of Nepal’s health system, culture, economy, and geography. The strategies for IMCI implementation included engagement of community and local stakeholders, engagement of partners in preparation and for implementation, integration of programs into existing structures, pilot testing, data-driven adaptations, and adaptation including of existing training, guidelines, and programs to fit the local context (Table 2).

**Table 2:** Implementation strategies used to implement facility- and community-based integrated management of childhood illness in Nepal, contextual factors, and implementation outcomes

**Outcome**	**Strategy**	**Examples of contextual factors**	**Results**
**Acceptability**	Training and orientation of mothers’ groups, local NGOs, traditional healers, and other community groups with the CB-IMCI guidelines; Use of FCHVs selected by local community (CB-IMCI).	Community health system and structure (facilitators)	Care-seeking for diarrhea, pneumonia, and fever for children under 5 rose to or nearly to 50% by 2014.
**Feasibility**	Local research; Pilot testing; Integration into existing district structure; Partner engagement for training and other implementation support (both FB- and CB-IMCI). Use of FCHVs and other community-based health workers to implement community-based care (CB-IMCI). Integration of CB-NCP into existing CB-IMCI program to create combined CB-IMNCI; Use of FCHVs for care delivery (CB-IMNCI).	Health system strength (both facilitator and barrier) Culture of data use; Prioritization of local research; Community health system and structure; Culture of donor and partner coordination (facilitators) Geography (barrier)	By 2009, IMCI had been implemented in all 75 districts. Scale-up occurred over 10 years, with expansion beginning in districts which already had community-based health programs in place. Use of existing FCHVs helped to reduce cost of program implementation and expansion.
**Fidelity**	Adaptation of existing standard WHO-IMCI training materials for Nepal’s specific needs and translation into Nepali language (both). Monitoring and supervision meetings between FCHVs, health facility supervisors, district health officers, and NGO trainers occur in the community and at health facilities (CB-IMCI).	Culture and capacity of data use; Prioritization of local research; Community health system and structure (facilitators)	An assessment of the IMCI program in 2017 found 30% of facilities reported stockouts in the previous 3 months [33]. Further, only 65% of facility health workers were found to have been trained in IMCI.
**Effectiveness and Reach**	Training using a cascade (training of trainers) system to reach providers from the central MOH level to the District Health Office (the local leads) to the facility to the community; Supervision integrated into District Health Offices (both); Adaptation to include neonatal interventions (both).	Community health system and structure (facilitator) Geography (barrier)	By 2009, IMCI had been implemented in all 75 districts. Care-seeking for children under 5 between 2001 and 2016 increased: for diarrhea, from 21 to 64%; for fever from 24 to 80%. In 2009, more than half of U5s received care for pneumonia or diarrhea from FCHVs [34].

*CB* Community-based, *FB* Facility-based, *FCHV* Female community health volunteer, *IMCI* Integrated management of childhood illness, *MOH* Ministry of Health, *NCP* Newborn care package, *NGO* Non-governmental organization, *U5* Under 5

#### Exploration (E)

Nepal’s Ministry of Health (MOH) established the Control of Diarrheal Diseases (CDD) program in 1982 in health facilities. In 1987, Nepal started the Acute Respiratory Infection Program, also facility-based. Recognizing that referral was not always a feasible option for children in remote districts due to geographic inaccessibility of health centers, during the mid-1990s, community health workers (CHWs) – specifically village health workers and maternal child health workers – and volunteers began to be assigned the responsibility of treating the diarrhea and pneumonia cases in the community. Despite the positive outcome, the MOH felt the need to assess the community-based care because of the concern about the quality of village health workers, maternal child health workers, and female community health volunteers (FCHVs). After years of policy discussion, Nepal pilot tested (strategy) community-based ARI care in four districts. Two districts followed a referral model (identification in the community and referral to facilities) and two followed a treatment model (diagnosis and treatment in the community using cotrimoxazole by the FCHVs, village health workers, and maternal child health workers). The treatment model was more effective and popular, with negligible overuse of antibiotics [26]. According to one KI, *“They do [a] pilot study and if those models are successful then we try to replicate in other parts of the country, and there are so many researches, those carried out in a small scale and later on replicated. For example CB-IMCI program, which was started from few pilot districts, but now it’s throughout the country.”*

#### Preparation (P)

In 1995, the MOH formed an IMCI working group with representatives from different divisions and centers within MOH, hospitals, and development partners. Strategies in this stage included pilot testing, strong stakeholder engagement, intersectoral collaboration, and committed national leadership to address child health problems. From 1997 through 1999, FB-IMCI was piloted in Mahottari district, with an evaluation meeting mid-1998 recommending its expansion, reflecting evidence-based decisions (strategy). In 1999, the community version was added: FCHVs were thought to be instrumental in the community to help reduce morbidity and mortality among children aged 2 months to 5 years. This combined facility- and community-based IMCI, an early approach to integration (strategy), was piloted in three more districts in 1999 and 2000 [26].

#### Implementation (I)

After successful piloting, intensive trainings were organized for health facility workers, reflecting a strategy of a cascade training system from district health offices to subdistrict health facilities. There were some challenges using the district health offices in training, such as frequent movement of the health workers. However, the strategy of district-level training created a sense of strong ownership of activities by the district involved. Training initially took 11 days, but after an evaluation suggested a similar level of knowledge and skills could be generated in seven days it was shortened [35]. In 2009, the IMCI program launched nationwide.

#### Adaptation (A)

In 2004, following the publication of studies in India and Bangladesh on community-based neonatal care programs, the MOH funded pilot projects for a similar program through its Neonatal Health Strategy [36]. From 2005 to 2009, the Morang Innovative Neonatal Intervention Program, a community-based pilot program implemented in Morang district, studied FCHVs’ ability to treat and refer neonatal infections, perform prenatal and postnatal visits, and offer newborn care counseling as part of a community-based newborn care package (CB-NCP). Following the introduction of this program, treatment for neonatal sepsis in the intervention district rose from 3% to 75% [37]. The decision was made to scale up this program, and a 2012 evaluation in 10 program districts found improvements in messaging to mothers around essential newborn care, but that strategies aimed at reducing mortality due to low birth weight or newborn asphyxia were less successful than expected [38].

Building on the work to reduce neonatal mortality, in late 2014, neonatal care was formally added to Nepal’s IMCI program and CB-IMCI became Community-Based Integrated Management of Neonatal and Childhood Illness (CB-IMNCI). The merging of the programs was intended to reduce management and service delivery overlap and challenges [39]. Nepal was comparatively early in its adaptation to include prioritized neonatal management in its national policies and programming (strategies).

#### Sustainment (S)

By 2009, IMCI had expanded nationally. Supervision and monitoring mechanisms were strengthened and use of FCHVs was an important basis of sustainment for both CB- and FB-IMCI programs. However, the 2015 MOH Annual Report recognized issues with the quality of FCHV performance, with further issues related to quality and coverage in IMNCI services [39]. A number of KIs worried about sustainability challenges of the program, citing overburdened FCHVs. A 2017 assessment of Nepal’s IMNCI program, after the study period, found all FCHVs interviewed had completed registers, conducted monthly mother’s group meetings in their respective communities, and were completing monthly reports for their health facility [33]. Improvements to on-site supervision and training, supply chains, and quality improvement mechanisms could further help to sustain the CB-IMNCI program.

### Contextual factors contributing to or hindering EBI implementation including IMCI

Important facilitating factors to IMCI implementation included existing community health system and structure, culture of data use, economic development, female empowerment, focus on universal health care and equity through national leadership, coordination of donors and implementers through a Sector Wide Approach (SWAp), evolution from vertical project-based funding to a horizontal approach to support the health sector work, engagement of the private sector to increase access, and prioritization and capacity of research done in Nepal (Table 3). Health systems strengthening was both a facilitator and a barrier, while geography was a barrier. Conflict was, surprisingly, neither a barrier nor a facilitator (see also Appendix 4). More details can be found in the full case study [40].

**Table 3:** Additional contextual factors associated with implementation of evidence-based interventions to decrease amenable under-5 mortality in Nepal

**Contextual Factor**	**Facilitator, Barrier, Both, or Neither**	**Description**
Economic development	Facilitator	Nepal’s steady economic growth during the study period was identified as an important contributor to health sector successes. Key informants observed that a growing number of Nepalese people working overseas improved economic status and increased financial access to health care through remittances; further, their exposure to other health care systems led families to have higher expectations of care and demand better quality
Female empowerment	Facilitator	During the study period efforts to increase female empowerment included education, addressing poverty, improving asset ownership, increasing women’s economic rights, and targeting women for microcredit programs to increase financial opportunities. In the health sector, efforts included government-sponsored village mothers’ groups, and granting authority and importance to FCHVs in communities
Focus on universal health care and equity through national leadership	Facilitator	Nepal committed to access to health care as a fundamental right of the people, prioritizing gender equality and social inclusion in health policy and delivery through policies including its Second Long Term Health Plan (1997–2017) which prioritized Nepal’s most vulnerable groups including women and children, rural populations, and underprivileged and marginalized people [41]. Following the people’s movement of 2006, a free health care policy was introduced in 2006 which provided essential health care services free of charge to the poor, disabled, elderly, and FCHVs up to primary healthcare centers and 25-bed hospitals. The following year this was expanded to all citizens at the health post level [42]. The 2007 interim constitution of Nepal enshrined health as a fundamental human right [43], and free basic primary care was extended to all citizens between 2008–11 – the same time period as the national IMCI rollout [41, 44]
Health system strengthening	Both	Nepal’s work to strengthen health systems broadly, including efforts to address geographic access, infrastructure, and human resources for health, was essential for facilitating IMNCI implementation and U5M reduction EBIs more generally. For example, the ability to integrate new IMNCI programming into existing community-level health systems structures was important its successful scaling across the country. However, health system strength was also a barrier to EBI implementation, such as human resources in more remote areas which grew at a slower pace compared to national increases. While the country made efforts to meet these needs, this left goals for training workers unmet, for example challenging the ability to achieve a goal of 7,000 trained maternal health workers by 2015
Conflict	Neither	Surprisingly, this was not a major barrier to EBI implementation including IMCI. While Nepal experienced a decade of armed conflict between the Government of Nepal and the Maoist insurgency between 1996 and 2006, neither side disrupted access to health services, and KIs reflected on policies which encouraged ongoing primary care. Most key health metrics including EBI implementation improved during this time, including U5M, vaccination rates, and antenatal care visits. Much of the pilot work for Nepal’s IMCI programming took place during this period

*EBI* Evidence-based intervention, *FCHV* Female community health volunteer, *KI* Key informant, *IMCI* Integrated management of childhood illness, *IMNCI* Integrated management of neonatal and childhood illness, *U5M* Under-5 mortality

#### Strong preexisting community health system and structure including community health workers

Within the preexisting community health system, FCHVs played an important role in health promotion and education, distribution of health commodities, and treatment and referral services for EBIs including IMCI [45]. Several KIs acknowledged FCHVs’ commitment and importance in delivery and acceptance of key EBIs needed for U5M reduction. Their responsibilities evolved beyond provision of reproductive health services, and facilitated the strategy of integrating maternal, neonatal, and child health-related initiatives, including CB-IMNCI work. Some challenges related to the increasing scope of their work and quality of care delivered were also noted. According to one KI, *“The most important thing behind FCHVs are that they are selected by the local community…They are regularly in touch and are the bridge between the health system and the community.”* However, concerns were identified for the sustainability of this facilitating factor reflecting overburdened FCHVs.

#### Culture of data use and prioritization of local research

Many KIs emphasized the importance of a culture of prioritizing local research (research conducted in Nepal by Nepal-based researchers) facilitated by existing in-country capacity, considered to be essential for Nepal to increase acceptability and appropriateness before implementing any intervention. For example, evidence produced by pilot programs for community-based treatment of ARI encouraged the MOH to support its expansion nationwide and eventually fold this into the national IMCI program. This was also the case with the early decision to expand neonatal care at the community level.

#### Geography

**Fig. 1:**
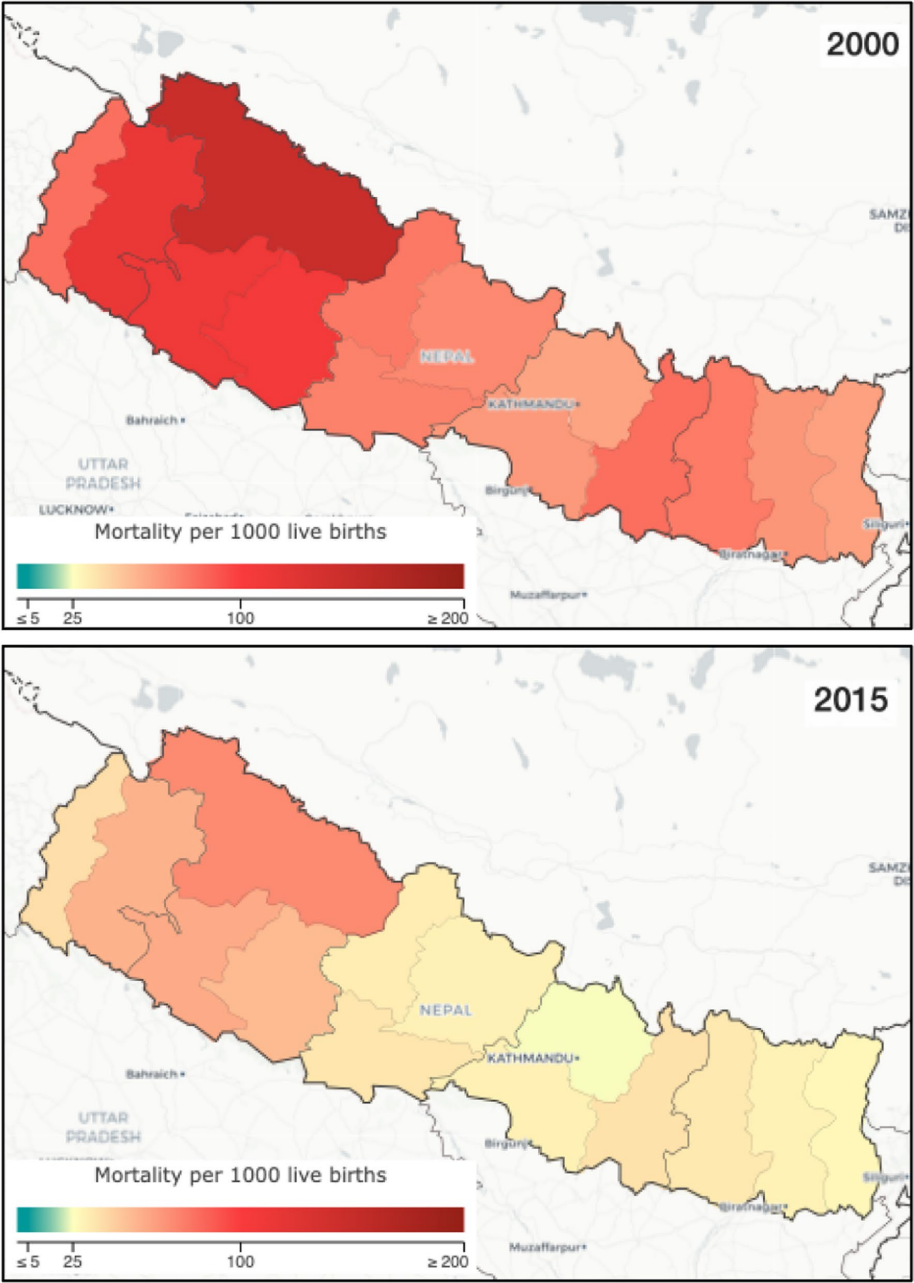
Under-5 mortality in Nepal’s 14 zones in 2000 and 2015. Source: Institute for Health Metrics and Evaluation (IHME). Local Burden of Disease – Under-5 mortality. Seattle, WA: IHME, University of Washington, 2019. Available from http://vizhub.healthdata.org/lbd/under5

Nepal’s geography remained a major challenge in the work to reduce U5M due to inequitable access to health care facilities. Despite efforts from the Government of Nepal, remote areas remain challenged in access, shown by persisting inequities in coverage and outcomes (Fig. 1) [46]. However, Nepal addressed this barrier through strategies to expand community-level care in hard-to-reach areas, for example training FCHVs to provide skilled delivery assistance so that women would not lose time in long-distance transport to facilities.

### Transferable lessons

We identified a number of transferable lessons through this work that are of potential interest to other countries working to decrease U5M generally through strategies to expand health systems EBIs and specifically through IMCI programs (Table 4). Lessons include developing a strong community health worker program with the establishment of CHWs, and a national focus on other factors important to reducing U5M though not explicitly health-related, such as improved roads and access to education.

**Table 4:** Transferable lessons and examples for other countries

**Lesson**	**Example**
Plan for equity from the beginning	Taking account of variations in access to facilities-based health care due to geography, Nepal established a strong community-based health program supported by CHWs
Develop a community health worker program	The FCHV program was expanded to include increased services rather than establish new cadres for each new community health service
Build on existing health system capacity by integrating new projects while simultaneously strengthening the underlying system	Nepal built on its existing FCHV program to expand its IMCI program to include neonatal care, while also working to engage with communities to increase demand and improve supply from facilities and in communities
Engage stakeholders including donors, implementing partners, and community members	This was important to increasing acceptability of EBIs and the ability to scale projects. Community engagement ensured participation of communities in program implementation, for example increasing comfort with FCHVs who were selected by village mothers’ groups
Address other factors related to U5M	Strengthening other sectors including safe water and sanitation, access to education, and improved infrastructure, all had important direct and indirect benefits to reducing U5M

*CHW* Community health worker, *EBI* Evidence-based intervention, *FCHV* Female community health volunteer, *IMCI* Integrated management of childhood illness, *U5M* Under-5 mortality

## Discussion

We used a hybrid implementation research framework to understand how Nepal implemented IMCI as an example of health system-delivered EBIs designed to contribute to the reduction of amenable U5M between 2000–2015. We found that the country chose implementation strategies which often either addressed barriers or leveraged facilitating contextual factors. These strategies included engagement of community and local stakeholders, engagement of partners in preparation and for implementation, integration of programs into existing structures, and pilot testing. Following steps from exploring the need, preparing, and pilot testing, IMNCI programming was implemented nationwide. This work was also facilitated by contextual factors including culture and capacity of data use, prioritization of local research, and existing community health system and structure.

Implementation research has long recognized that adaptation is necessary to achieve impact of EBIs across settings – but often research does not specify whether adaptations were made to planned EBIs [8]. Nepal deployed adaptation as a critical strategy and step in the implementation pathway. It made adaptations of strategies based on local research and available health systems and structure; adaptations to local context to leverage its existing FCHVs, culture of data use and local research capacity, as well as to face the barrier of geographical challenges; and adaptations targeting implementation outcomes, to improve reach and effectiveness. The government expanded FCHVs’ roles as community providers for CB-IMCI, adapted and expanded WHO training materials and guidelines for FB-IMCI and CB-IMCI, and eventually adapted the program again to include the neonatal component. These were data-driven adaptations including of existing training, guidelines, and programs to fit the local context. They were not always successful however, and there has been debate about whether the adaptation to IMNCI was the right call given lack of training of FCHVs, low demand from families, and existence of other programs that may have been a better fit [47]. A study of IMNCI implementation in India found that increased supervision and standardization of training were important for CHWs given the added newborn services [48], further adaptations that may be necessary in Nepal to increase the quality and sustainability of its IMNCI programming.

Nepal’s investment in community- and facility-based IMCI implementation was successful in part because of its leveraging of an important facilitating contextual factor: the existing strong cadre of community-based healthcare champions known as female community health volunteers. This finding is supported by others who found that community health interventions implemented with FCHVs had high coverage during the study period[49].

Yet the FCHV system has also began to experience strains, related to expansions to its scope of activities and an aging workforce. Increases in program demands, service quality issues, and growing access of the population to alternative sources of health care as the country addressed geographic and financial barriers have led to concerns around the sustainability of this system that will need to be attended to[45, 50]. Duplications across what FCHVs provide at the community level by different divisions contributes to overburdening of FCHVs who might be required to perform tasks supporting multiple program activities [49], in a context of insufficient supervision and training, found to have historically been challenges in Nepal [51]. This experience of growing pressure on Nepal’s core of FCHVs is reflected in literature on the experiences of CHWs in health systems elsewhere [52–54]. WHO recommendations have been updated to further institutionalize the role of CHWs through salary and supervision recommendations and additional steps to ensure CHWs operate in an official capacity including certification, community engagement, and mobilization of community resources to more firmly anchor CHWs as a community resource [55]. In recognition of these challenges, the MOH has partnered with local researchers to trial a program that will inform the development of a new CHW system, including through professionalization of the workforce and improving the quality of service delivery [56].

The culture of data use and leveraging existing local research capacity were important contextual factors to facilitate pilot programs such as the Morang Initiative Neonatal Intervention program. This pilot ultimately led to Nepal’s adaptation to include neonatal care provision in its IMCI program. Similar to our findings, evidence from IR studies in several countries found that policymakers valued local implementation research and that international research evidence was found useful for identifying policy options that were relevant in local settings [7]. Other studies of Nepal’s work to reduce U5M found the government to have been progressive in the adoption of EBIs, and has been important in fostering the culture of data use to inform program design [57]. However, the culture of data use was also associated with some challenges. While prioritization of local research has been a facilitator to the country’s successful implementation of many EBIs, it has also caused some delays between decisions to explore a new EBI and the actual nationwide scaling of an intervention. Further, some EBIs found to be successful in local pilots were never scaled in the course of the study period [40]. Finding a balance of commitment to local research and decisions to adopt national rollout of interventions based on existing evidence could have an impact on U5M outcomes in the future.

The Government of Nepal has been working towards universal health coverage (UHC) through expanded social insurance and free primary health care programs in recent years. This leadership and vision of health care as a fundamental right has persisted across national political transitions, and in 2016 Nepal introduced the National Health Insurance Program [58]. A driving goal of Nepal’s UHC expansion has been to increase access in health policy and delivery – supporting its equity strategy [59]. While ensuring equity was a core strategy, and national coverage increased for many of the IMCI markers and metrics between 2001 and 2016, there was not always uniform benefit across urban and rural areas and across wealth quintiles. These differences demonstrate that there are opportunities to improve equity between groups while continuing to make substantial progress for the population as a whole. Thapa and colleagues looked at equity and coverage of many of the leading U5M EBIs in Nepal between 2001 and 2016 and noted that as coverage increased inequitable coverage decreased steadily – if slowly – across many EBIs [60]. There are examples of more effective strategies to reduce inequities across income and geography, including Nepal’s immunization program, which has made continuous ongoing outreach efforts to ensure an uninterrupted supply of vaccines for children under 5 [60].

We found other important factors which facilitated implementation of EBIs including IMCI and contributed to Nepal’s dramatic drop in U5M included those less explicitly focused on health care-targeted preventative and curative efforts. These include improvements to infrastructure and communications – such as to roads, schools, electricity, and communication technology; addressing social determinants and other contributors to U5M – such as water, sanitation, and hygiene (WASH); and access to family planning, as well as women’s education and empowerment through initiatives such as expanding economic opportunities and increasing asset ownership. A study of success factors in countries on track to meet MDG goals for U5M found non-health system investments responsible for as much as half the U5M reductions [61]. Infrastructure and other non-health system investments can contribute to the momentum needed in countries working to accelerate progress in health outcomes.

Despite its successes, challenges remain for Nepal. Overcoming service gaps and equity barriers that result from the country’s difficult geography is an important persistent challenge as most areas in the country are hills and mountains. Strengthening local service provision has happened but ongoing gaps in access and coverage remain. Adapting existing programs that have been successful in the past, such as the system of FCHVs, to be sustainable going forward will be a critical undertaking.

This paper has a number of limitations. Key informant interviews took place after the period of interest, and so results depended on recall about strategies and contextual factors as well as the pathway to implementation dating back to 2000–2015. Resources and timing of the study also limited the range of key informants, so we were not able to interview frontline implementers or communities about the time period of interest. Due to limitations of the research scope we did not include all potentially relevant contextual factors including those adjacent to or beyond the health system. Data availability and quality limited findings on implementation outcomes as key factors such as cost, quality, and reasons for variability in outcomes were often absent in the published reports and quantitative data. The limitations on data availability meant that we were unable to include subnational variability on implementation outcomes and context, an area for further work. The design of this work was specifically focused on the implementation of known EBIs in a real-world setting. As such the attribution of specific EBIs to overall amenable U5M reduction was not directly measurable.

## Conclusion

Using implementation research we broadened understanding of how and why Nepal used a number of chosen implementation strategies to implement IMCI as an EBI known to reduce amenable U5M in LMICs. This approach was important in overcoming the know-do gap which has hindered progress in achieving child health goals, through adaptation, sustainment, and outcomes beyond effectiveness. The importance of understanding context and then choosing strategies that leverage facilitating factors such as community health programs, culture of data use, and leadership, and ones which address barriers such as geography, are useful lessons for countries working to accelerate expansion of quality EBIs to continue work to achieve child health targets.

## Data Availability

The Demographic and Health Surveys data used in the current study are available from the DHS website. Nepal DHS 2001: https://dhsprogram.com/publications/publication-FR132-DHS-Final-Reports.cfm Nepal DHS 2016: https://dhsprogram.com/publications/publication-fr336-dhs-final-reports.cfm The Institute for Health Metrics and Evaluation (IHME) Low- and Middle-Income Country Neonatal, Infant, and Under-5 Mortality Geospatial Estimates 2000–2017 Local Burden of Disease data used in the current study are available for download from IHME: http://ghdx.healthdata.org/record/ihme-data/lmic-under5-mortality-rate-geospatial-estimates-2000-2017. https://doi.org/10.6069/9ABZ-XG84 The case study that is the basis for this article can be found here: https://www.exemplars.health/-/media/files/egh/resources/underfive-mortality/nepal/nepal-case-study_-final-_10042020.pdf Data access is restricted to users with appropriate ethics approval from the committees listed in the Ethical Considerations section. A reader or reviewer may apply to the authors for access by providing a written description of background, reasons, and intended use. If the methodology does not violate the condition of informed consent under which the interview was conducted, and the proposal approved by UGHE and other relevant ethics boards, the user can obtain the data from the corresponding author, and include one of the authors in the project and analysis.

## Appendix 1

Hybrid framework for understanding health system-delivered evidence-based interventions to reduce under-5 mortality in low- and middle-income countries. *The authors have obtained permission from the University of Global Health Equity (UGHE) to include this figure.*

## Appendix 2

Tables 5 and 6

**Table 5:** Infant and child under-5 mortality evidence-based interventions

**Cause of Death**	**Evidence-based intervention**	
**Lower respiratory infections**	Antibiotic treatment	
	Vaccination: PCV	
	Vaccination: Hib	
	Community-based management	
	Facility-based management	
**Diarrheal diseases**	Oral rehydration therapy	
	Zinc supplementation	
	Vaccination: Rotavirus	
	Community-based management	
	Facility-based management	
**Malaria**	Antimalarial combination therapy	
	Rapid diagnostic testing	
	Insecticide-treated nets	
	Indoor residual spray	
	Intermittent preventative therapy for high-risk groups	
	Community-based management	
	Facility-based management	
**Measles**	Vaccination: Measles	
	Vitamin A supplementation (prior to vaccination)	
**Malnutrition**	Exclusive breastfeeding for six months	
	Continued breastfeeding and complementary feeding after six months	
	Vitamin A supplementation	
	Management of severe acute malnutrition (ready-to-use food, rehydration, antibiotics)	
**HIV**	ARV treatment for infants and children	
	HIV testing of children born to HIV + mothers	
	Prevention of mother-to-child transmission	Early diagnosis of pregnant women (or pre-pregnancy)
		PMTCT treatment for mothers* and post-partum to exposed infants
		Elective C-section for untreated HIV + mothers**; replacement feeding**
		ARV treatment for mother for life as prevention (started in 2012)
		Exclusive breast feeding
**Meningitis**	Vaccination: PCV meningococcal	
	Vaccination: Hib	
	Vaccination: Meningococcal	
	Antibiotic treatment	
	Chemoprophylaxis during acute outbreaks	
**Other vaccine preventable diseases**	Vaccination: Tetanus	
	Vaccination: Diphtheria	
	Vaccination: Pertussis	
	Vaccination: Polio	

**Table 6:** Neonatal mortality evidence-based interventions

**Period of Risk**	**Evidence-based intervention **	
**Preconception**	Folic acid supplementation	
**Antenatal**	Tetanus vaccination	
	Malaria prevention and treatment	Intermittent presumptive treatment
		ITNs
	Iodine supplementation (in endemic iodine deficient settings)	
	4 or more antenatal visits (ANC4)	
	Prevention and treatment of preeclampsia and eclampsia	Calcium supplementation*
		Low-dose aspirin for high-risk women*
		Antihypertensive treatment for severe hypertension
		Magnesium sulfate
		Early delivery
**Intrapartum**	Antibiotics for PPROM	
	Corticosteroids for preterm labor	
	C-section for breech or obstructed labor	
	Active management of delivery (including partograph)	
	Clean delivery practices (incl. clean cord-cutting)	
	Trained birth attendant	
	Facility-based delivery	
	Basic emergency obstetric and newborn care (BEmONC)	
	Comprehensive emergency obstetric and newborn care (CEmONC)	
	Timely transport for higher level care for mother	
**Postnatal**	Newborn resuscitation	
	Immediate breastfeeding	
	Prevention and management of hypothermia	Immediate drying and wrapping
		Delayed bathing
		Skin-to-skin
		Baby warming
	Kangaroo care for LBW/prematurity	
	Timely transport for higher level care for mother	
	Post-partum visits to identify danger signs and provide active referral	
	Antibiotics for suspected or confirmed infection	
	Surfactant therapy for respiratory distress syndrome and prematurity	
	Neonatal intensive care units (equipped, trained staff, standards and protocols established and followed)	

## Appendix 3

Table 7

**Table 7:** Change in under-5 and neonatal mortality and health system evidence-based interventions to lower amenable under-5 mortality, in overall rate and by subpopulation, between 2001 and 2016 in Nepal.

	**Year**	**National**	**Rural**	**Urban**	**Equity gap (absolute difference)**	**Lowest wealth quintile**	**Highest wealth quintile**	**Equity gap (absolute difference)**
U5M (per 1000 population)	2001	91	112	66	46	155	82	73
	2016	39	44	34	10	74	35	39
NMR (per 1000 population)	2001	39	49	37	12	57	48	9
	2016	21	26	16	10	36	19	17
U5 death from respiratory infections	2001	14,577	N/A	N/A	N/A	N/A	N/A	N/A
	2016	4,644	N/A	N/A	N/A	N/A	N/A	N/A
U5 death from diarrheal disease	2001	6,408	N/A	N/A	N/A	N/A	N/A	N/A
	2016	1,005	N/A	N/A	N/A	N/A	N/A	N/A
Children U5 with symptoms of ARI 2 weeks preceding survey (%)	2001	23	23	24	1	N/A	N/A	N/A
	2016	2	3	2	1	3	1	2
Care-seeking for ARIs (%)*	2001	24	23	33	10	19	33	14
	2016	85	81	90	9	N/A	N/A	N/A
Children U5 with diarrhea 2 weeks preceding survey (%)	2001	20	21	17	4	N/A	N/A	N/A
	2016	8	7	8	1	6	7	1
Oral rehydration therapy (either ORS or increased fluids) (%)	2001	46.5	46	63	17	38	58	20
	2016	53	48	58	10	55	74	19
Care-seeking for diarrhea (%)	2001	21	21	23	2	15	22	7
	2016	64	70	60	10	55	59	4
Children U5 with fever 2 weeks preceding survey (%)	2001	32	32	27	5	N/A	N/A	N/A
	2016	21	19	23	4	18	22	4
Care-seeking for fever (%)*	2001	24	23	33	10	19	33	14
	2016	80	76	82.5	6.5	59	84	25

Source: Nepal Demographic and Health Surveys 2001 and 2016, Countdown to 2030, and Institute for Health Metrics and Evaluation [6, 18, 27, 62].

*ARI* Acute respiratory infection, *NMR* Neonatal mortality rate, *ORS* Oral rehydration solution, *U5* Under 5, *U5M* under-5 mortality.

*in the 2001 DHS, the rates for care-seeking for ARIs and fever were combined but separated in the 2016 DHS.

N/A: data not disaggregated by category.

## Appendix 4

Table 8

**Table 8:** Contextual factors identified in the Nepal Exemplars in Under-5 Mortality case study and those identified as being relevant for the integrated management of neonatal and childhood illness intervention

**Contextual factors**	**Relevant to IMNCI**
Addressing social determinants and other contributors to U5M	
Conflict	X
Culture of data use	X
Donor and international partner resources and coordination	
Economic growth and development	X
Female community health volunteers	
Focus on universal health care and equity: national leadership and commitment	X
Geography	X
Health financing	
Health system strengthening	X
Improved access to family planning, increased birth spacing, and decreased fertility rates	
Infrastructure and communications	
Prioritization of local research	X
Preexisting community health system and structure including community health workers	X
Quality assessment/improvement	
WASH	
Women’s education/empowerment	X

*IMNCI* Integrated management of neonatal and childhood illness; *WASH* water, sanitation, and hygiene.

## Abbreviations

ARI: Acute respiratory infection
CB-IMCI: Community-based integrated management of childhood illness
CB-IMNCI: Community-Based Integrated Management of Neonatal and Childhood Illness
CB-NCP: Community-Based Newborn Care Package
CDD: Control of Diarrheal Diseases
CHW: Community health worker
DHS: Demographic and Health Surveys
EBI: Evidence-based intervention
EPIAS: Exploration, Preparation, Implementation, Adaptation, and Sustainment
FB-IMCI: Facility-based integrated management of childhood illness
FCHV: Female community health volunteer
Hib: Haemophilus influenzae type B vaccine
IHME: Institute for Health Metrics and Evaluation
IMCI: Integrated management of childhood illness
IR: Implementation research
KI: Key informant
LMIC: Low- and middle-income country
MDG: Millennium Development Goal
MOH: Ministry of Health
PCV: Pneumococcal conjugate vaccine
U5M: Under-5 mortality
UHC: Universal health coverage
WASH: Water, sanitation, and hygiene
